# ATF4 deficiency protects hepatocytes from oxidative stress *via* inhibiting CYP2E1 expression

**DOI:** 10.1111/jcmm.12166

**Published:** 2013-11-06

**Authors:** Chunxia Wang, Houkai Li, Qingshu Meng, Ying Du, Fei Xiao, Qian Zhang, Junjie Yu, Kai Li, Shanghai Chen, Zhiying Huang, Bin Liu, Feifan Guo

**Affiliations:** Key Laboratory of Nutrition and Metabolism Institute for Nutritional Sciences Shanghai Institutes for Biological Sciences Chinese Academy of Sciences, The Graduate School of the Chinese Academy of SciencesShanghai, China

**Keywords:** ATF4, primary hepatocytes, CYP2E1, oxidative stress, ROS, TG accumulation

## Abstract

Activating transcription factor (ATF) 4 is involved in the regulation of oxidative stress in fibroblasts and neurons. The role of ATF4 in hepatocytes, however, is unknown. The aim of this study was to investigate the role of ATF4 in hepatocytes in oxidative stress under a high-fat diet (HFD). Here, we showed that palmitate-stimulated reactive oxygen species (ROS) production and triglyceride (TG) accumulation is blocked by ATF4 deficiency in primary hepatocytes. Consistently, HFD-induced oxidative stress, TG accumulation and expression of cytochrome P450, family 2, subfamily, polypeptide 1 (CYP2E1) are also blocked by knocking down ATF4 expression in the mouse liver. This suggests that ATF4 might regulate oxidative stress *via*CYP2E1 under an HFD. In addition, we observed that expression of CYP2E1 is indirectly regulated by ATF4 in a cAMP-responsive element binding protein (CREB)-dependent manner, which can directly activate the CYP2E1 promoter activity. Notably, ATF4-stimulated ROS production is inhibited *in vivo* by treatment with diallyl sulphide, a selective CYP2E1 inhibitor. Finally, we showed that ATF4 expression in the liver is responsible for the protective effects against HFD-induced CYP2E1 expression, oxidative stress, and TG accumulation. Taken together, these observations suggest that ATF4 is a novel regulator of oxidative stress as well as accumulation of TG in response to HFD.

## Introduction

Activating transcription factor (ATF) 4 belongs to the family of basic zipper-containing proteins. It is expressed in a wide variety of tissues and is stimulated in response to various cellular stresses, such as amino acid deprivation and integrated stress stimulation 1–2. Activating transcription factor 4 is known to be involved in the regulation of various biological processes including long-term memory 3–4, osteoblast differentiation 5 and lipid and glucose metabolism 6. In addition, ATF4 has been shown to play an important role in the regulation of redox control. The effect of ATF4 in this regulation, however, is cell type-specific. For example, while ATF4-deficient fibroblasts are prone to oxidative stress induced by depletion of amino acids 7, recent studies indicate that ATF4 deficiency is protective against oxidative stress in neurons 8 or HEK293 cells 9. The effect of ATF4 on oxidative stress in hepatocytes, however, remains unknown.

Our previous studies indicated that ATF4 is a key regulator of lipid metabolism and thermogenesis 10, and ATF4 deficiency protects mice from high-carbohydrate diet-induced liver steatosis 11. Recently, Seo *et al*. observed that ATF4-null mice do not develop non-alcoholic fatty liver diseases (NAFLD) when induced by a high-fat diet 6. In this work, this phenotype was proved. Activating transcription factor 4 is a transcription factor of the unfolded protein response (UPR) 12. The UPR is activated in obesity-associated fatty liver disease and alcohol-induced liver injury, which are concomitant with steatosis, thus raising the possibility that endoplasmic reticulum (ER) stress-dependent alteration in lipid homoeostasis is the mechanism that underlies this steatosis. Failed ER stress adaptation possibly mediated through calcium perturbations or reactive oxygen species 13. Notably, ER stress can induce the expression of fibroblast growth factor 21 (FGF21) 14, and FGF21 has emerged as an important metabolic regulator of glucose and lipid metabolism. Fibroblast growth factor 21 reduced lipid levels and reversed hepatic steatosis, which was associated with FGF21 inhibition of nuclear sterol regulatory element binding protein-1 and the expression of a wide array of genes involved in fatty acid and triglyceride synthesis 15. High-fat diet normally induces oxidative stress 16–17, which is a major contributor to the development of NAFLD 18,19, and ATF4 is known to play a role in oxidative stress 7,8. However, while these occurrences are correlative, the underlying mechanisms remain unclear, and therefore the purpose of this study was to investigate this and elucidate any underlying molecular mechanisms. We observed that knock-down of ATF4 in the liver protects against HFD-induced oxidative stress and triglyceride (TG) accumulation both *in vitro* and *in vivo*. Moreover, we show that this effect can be mediated by inhibiting the expression of cytochrome P450, family 2, subfamily e, polypeptide 1 (CYP2E1), other than the regulation of lipid metabolism, ER stress, or FGF21 expression.

## Materials and methods

### Animal experiments

Heterozygous (*Atf4*^*+/−*^) mice on 129 SV background were kindly provided by Drs Tim Townes (Univ. of Alabama), Douglas Cavener (Penn State Univ.), and Bob Paulson (Penn State Univ.). *Atf4*^*+/−*^ mice were bred to produce homozygous (*Atf4*^*−/−*^) and wild-type (*Atf4*^*+/+*^) mice and genotyping was performed as previously described 21. Six-week-old male *Atf4*^*+/+*^ or *Atf4*^*−/−*^ mice were fed *ad libitum* for 16 weeks continuously either on a HFD or normal diet (ND) (Research Diet Inc., New Brunswick, NJ, USA). To study the effects of ATF4 in liver on CYP2E1 expression, 8-week-old male C57BL/J6 were injected with adenovirus expressing ATF4 (Ad-ATF4) or blank adenovirus (Ad-Null) through tail vein injection by using 1 × 10^9^ pfu/mice treated with or without diallyl sulphide (DAS, 200 mg/kg, i.p) once a day for 7 days. To study the effects of ATF4 in liver on HFD-induced oxidative stress and TG accumulation, 6-week-old male *Atf4*^*+/+*^ mice were fed a HFD for 1 week, then injected with adenovirus expressing dominant negative ATF4 (Ad-ATF4 DN) or control green fluorescent protein adenovirus (Ad-GFP), followed by continuing HFD feeding for 2 weeks. All mice were maintained on a 12-hr light/dark cycle under controlled temperature (25°C) with free access to water. At the end of the experiment, animals were killed by CO_2_ inhalation, and liver tissues were collected and frozen immediately in liquid nitrogen for further analysis. The ATF4 overexpression plasmid 22 was kindly provided by Dr. Zaiqing Yang (Huazhong Agricultural University, Wuhan, China). The plasmid of ATF4 dominant negative mutant (pEF/mATF4M) 23 was kindly provided by Dr. Alam J. (Yale Univ.). All animal experimental procedures were approved by the Institutional Animal Care and Use Committee of Institute for Nutritional Sciences (permit number: INS09-1001).

### Histological analysis of tissues

Liver samples were fixed in 4% paraformaldehyde overnight and stained with haematoxylin and eosin as previously described 24.

### Measurement of triglyceride (TG), malondialdehyde (MDA), superoxide dismutase (SOD), reduced glutathione (GSH), hydrogen peroxide (H_2_O_2_) and ROS in livers

Hepatic lipids were extracted with chloroform-methanol (2:1) according to Folch’s method 25. Hepatic TG, MDA, GSH, SOD and H_2_O_2_ were measured with triglyceride kit (Wako, Chuo-ku, Osaka, Japan), MDA, SOD and reduced glutathione kits (Jiancheng Biotechnology, Nanjing, China), and Amplex® Red Hydrogen Peroxide/Peroxidase Assay Kit (Invitrogen, Carlsbad, CA, USA), respectively, according to the manufacturer’s instructions. Intracellular ROS was measured with the fluoroprobe 6-carboxy-2′,7′-dichloro-dihydrofluorescin diacetate (H_2_DCF-DA, Molecular Probes, Portland, OR, USA) as described previously 26. Briefly, primary hepatocytes after various treatments were incubated for 30 min. in the dark at 37°C with 10 μM H_2_DCF-DA. After incubation with H_2_DCF-DA, cells were rinsed twice with PBS and harvested for an immediate detection of fluorescence intensity of DCF with FACscan flow cytometer (Becton Dickinson, Bohemia, NY, USA). The protein levels of each sample were determined with Pierce BCA Protein Assay reagent (Thermo Scientific, Rockford, IL, USA).

### Primary hepatocyte isolation, cell culture and treatments

Male *Atf4*^*+/+*^ or *Atf4*^*−/−*^ mice were anaesthetized by intraperitoneal (IP) injection with chloral hydrate. Primary hepatocytes were prepared by collagenase perfusion as described previously 27. Isolated hepatocytes were cultured in 10% FBS DMEM before initiating treatment. After attachment, primary hepatocytes from *Atf4*^*+/+*^ mouse were transfected with siRNA at a concentration of 40 pmol/l by using X-tremeGene siRNA Transfection Reagent (Roche Diagnostics, Mannheim, Germany), while the mock group was treated with equal volume of X-tremeGene siRNA Transfection Reagent. Primary hepatocytes were infected with the indicated adenovirus for 48 hrs. For palmitate treatment, primary hepatocytes were treated with 0.2 mM palmitate (+Pal) or vehicle (−Pal) for 24 hrs.

### Hepatocytes oxygen consumption

Hepatocytes oxygen consumption was measured with BD Oxygen Biosensor System plate (BD Biosciences, Sunnyvale, CA, USA) according to the manufacturer’s instructions. Plate was read with FlexstationII384 (Molecular Devices, Sunnyvale, CA, USA) at 3 min. intervals for 120 min. at an excitation wavelength of 485 nm and emission wavelength of 630 nm. The slope rate of the oxygen consumption curve during the test period was calculated.

### Generation and administration of recombinant adenoviruses

The recombinant adenoviruses used for ATF4 overexpression and ATF4 DN expression was generated by using AdEasy™ Vector System (Qbiogene, Carlsbad, CA, USA). Adenoviruses were purified by ultracentrifugation in caesium chloride gradient and then quantified. Viruses were diluted in PBS and administered at a dose of 10^7^ pfu/well in 12-well plate or through tail vein injection by using 1 × 10^9^ pfu/mice.

### Cell culture and treatments

HepG2 cells or primary hepatocytes were maintained in DMEM with 25 mmol/l glucose (Gibco, Invitrogen, Carlsbad, CA, USA), 10% FBS, 50 mg/ml penicillin and streptomycin at 37°C, and 5% CO_2_–95% air. For ATF4 overexpression, HepG2 cells were infected with adenovirus expressing ATF4 or GFP. For siRNA transfection, double-stranded siRNA targeting mouse ATF4 (sense 5′-GAGUUAGUUUGACAGCUAATT-3′, antisense 5′-UUAGCUGUCAAACUAACU-CCA-3′) was purchased from GenePharma (Shanghai, China).

### RNA isolation and relative quantitative RT–PCR

Total RNA was prepared from frozen tissues with TRIZOL (Invitrogen) reagent. Two microgram of RNA was reversely transcribed with random primer (Invitrogen) and M-MLV Reverse Transcriptase (Invitrogen). Quantitative amplification by PCR was carried out using SYBR Green I Master Mix reagent by ABI 7900 system (Applied Biosystem, Foster, CA, USA). GAPDH was used as an internal control for each gene of interests. The sequences of primers mainly used in this study are shown in Table 1.

**Table 1:** Primers for RT-PCR used in this work

Gene	Primers	Length amplified (bp)
*Atf4*	F 5′-TGACTTCGATGCTCTGTTTCGA-3′ R 5′-CCAACGTGGTCAAGAGCTCAT-3′	68
*Cyp2e1*	F 5′-TTCGGGCCAGTGTTCACA-3′ R 5′-GACAGCCTTGTAGCCATGCA-3′	69
*Chop*	F 5′-CCTAGCTTGGCTGACAGAGG-3′ R 5′-CTGCTCCTTCTCCTTCATGC-3′	196
*Sod1*	F 5′-GGACCTCATTTTAATCCTCACTCTAAG-3′ R 5′-TGCCCAGGTCTCCAACATG-3′	76
*Gpx1*	F 5′-CCACCGTGTATGCCTTCTCC-3′ R 5′-AGAGAGACGCGACATTCTCAAT-3′	105
*Gadph*	F 5′-TGTGTCCGTCGTGGATCTGA-3′ R 5′-CCTGCTTCACCACCTTCTTGAT-3′	77

### Western blotting

Whole-cell lysates from frozen tissues were isolated by using RIPA lysis buffer (150 mM Tris-HCl, 50 mM NaCl, 1% NP-40, 0.1% tween-20), and centrifuged at 15,300 ×*g* for 20 min. Then, the supernate was mixed with isometric sodium dodecyl sulphate (SDS) buffer [125 mM Tris hydrochloride (pH 6.8), 10% mercaptoethanol (vol/vol), 4% SDS (wt/vol), 20% glycerol (vol/vol), and 0.002% bromophenol blue]. The mixture was heated for 10 min. at 100°C. Supernatants were subjected to 10% SDS-PAGE gels and blotted by wet blotting. Primary antibodies including ATF4 (Santa Cruz Biotechnology, Santa Cruz, CA, USA), CYP2E1 (Millipore, Temecula, CA, USA), complex proteins I–III (Invitrogen) were incubated overnight at 4°C and visualized by ECL Plus (GE Healthcare, Buckinghamshire, UK). Band intensities were measured by using Quantity One (Bio-Rad Laboratories, Hercules, CA, USA) and normalized to ACTIN.

### Transient transfections and luciferase assays

A total of 293T cells were maintained in DMEM containing 10% FBS and transfections were performed with lipofectamine 2000 (Invitrogen) according to the manufacturer’s instruction. The pRL-SV40 (Promega, Madison, WI, USA) that carries renilla luciferase was also cotransfected as an internal control for monitoring the transfection efficiency. The vectors of CYP2E1 promoter (CYP2E1-P2600) and overexpression of ATF4 or CREB (pCMV-ATF4 or pCMV-CREB) were cotransfected into 293T, and pCMV-HA (control) as control. Forty-eight hours later, cells were harvested, and luciferase activities were analysed by using the Dual-Luciferase assay kit (Promega) according to the manufacturer’s instruction. Values were expressed as relative to the control Renilla to exclude the effects of the differences in transfection efficiency.

### ChIP assays

ChIP assays were performed according to the manufacturer’s protocols (Millipore, Billerica, MA, USA). Briefly, the DNA-protein complexes in 293T cells were cross-linked with 1% formaldehyde. Cells were lysed and sonicated prior to immunoprecipitation with anti-CREB antibody (Cell Signaling, Beverly, MA, USA), anti-ATF4 (Santa Cruz Biotechnology), or normal rabbit IgG (Santa Cruz Biotechnology) as a negative control at 4°C overnight. DNA-protein immunocomplexes were collected by using protein G magnetic beads. An immunoprecipitated CYP2E1 promoter was quantified by using PCR with primers designed to amplify the region encompassing the 130 bp containing the CRE site (forward, 5′-CCAGGGCACGCGGGTCCCGC-3′ and reverse, 5′-TGGTGGGGTG-AGAACAGG-3′) or the region encompassing the 100 bp containing the CRE site (forward, 5′-TGGTGATTCAGGTACTACTG-3′ and reverse, 5′-ACCACCTCTAGA-CACGGTAC-3′).

### Data analysis

All data are expressed as means ± SEM, except for specific indication. Significant differences were assessed either by two-tailed Student’s *t*-test or one-way anova followed by the Student-Newman-Keuls (SNK) test. *P* < 0.05 was considered statistically significant.

## Results

### ATF4 deficiency protects from palmitate-induced oxidative stress and TG accumulation in primary hepatocyte culture

**Figure 1:**
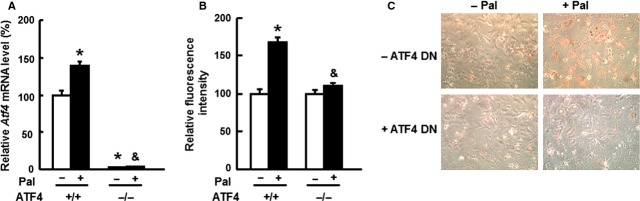
ATF4 deficiency protects primary hepatocytes from palmitate-induced ROS production and TG accumulation. Primary hepatocytes isolated from *Atf4*^*+/+*^ (ATF4 + /+) and *Atf4*^*−/−*^ (ATF4−/−) mice were treated with 0.2 mM palmitate (+Pal) or vehicle (−Pal) for 24 hrs, *Atf4*mRNA levels (A) and ROS production (B) were then compared. Primary hepatocytes isolated from *ATF4*^*+/+*^ mice were infected with adenovirus expressing an ATF4-dominant negative mutant (+ATF4 DN) or control green fluorescent protein adenovirus (−ATF4 DN) for 24 hrs, then treated with 0.2 mM palmitate (+Pal) or vehicle (−Pal) for 24 hrs. TG accumulation was stained with Oil red O (C). * indicates *P* < 0.05 as compared with the *Atf4*^*+/+*^/−Pal group; & indicates *P* < 0.05 as compared with the *ATF4*^*+/+*^/+Pal group. Data are the means ± SEM of at least two independent experiments (*n* = 4–6).

To test the possibility that deletion of ATF4 is protective against oxidative stress in hepatocytes, ATF4 expression was examined following palmitate treatment in primary hepatocytes isolated from both *Atf4*^*+/+*^ and *Atf4*^*−/−*^ mice. Palmitate has been shown to induce oxidative stress and ROS production 28 and TG accumulation 29, *Atf4* mRNA levels significantly increased with palmitate treatment in the primary hepatocytes isolated from *Atf4*^*+/+*^ mice, but not in *Atf4*^*−/−*^ mice (Fig. 1A). Moreover, there was no significant difference in ROS production between untreated primary hepatocytes isolated from *Atf4*^*−/−*^ mice as compared with *Atf4*^*+/+*^ mice (Fig. S1). Consistent with these results, palmitate-induced ROS production and TG accumulation were also blocked by ATF4 deletion (Fig. 1B and C). These results indicated that ATF4 is a pro-oxidative stress factor in primary hepatocyte cultures.

### ATF4 deficiency protects mice from HFD-induced oxidative stress and TG accumulation

**Figure 2:**
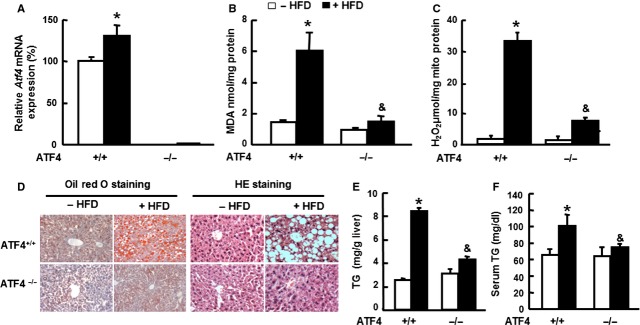
ATF4 deficiency protects mice from oxidative stress induced by high-fat diet (HFD). Six-week-old male *Atf4*^*+/+*^ (ATF+/+) and *Atf4*^*−/−*^ (ATF−/−) mice were fed HFD (+HFD) or normal diet (−HFD) for 16 weeks (*n* = 6). Expression of *Atf4*mRNA (A), levels of hepatic MDA (B), and H_2_O_2_ in hepatic mitochondria (C) was compared between the two strains. Representative histological analysis of livers from *Atf4*^*+/+*^ and *Atf4*^*−/−*^ mice (D), the left panel was oil red O staining (magnification 100 × ), and the right panel was stained with haematoxylin and eosin. The contents of TG in liver (E) and serum (F) in *Atf4*^*+/+*^ and *Atf4*^*−/−*^ mice were compared between the two strains. * indicates *P* < 0.05 as compared with the *Atf4*^*+/+*^/−HFD group; & indicates *P* < 0.05 as compared with the *Atf4*^*+/+*^*/*+HFD group. Data are the means ± SEM of two independent experiments.

To investigate whether ATF4 is also important for regulating oxidative stress and TG accumulation *in vivo*,*Atf4*^*+/+*^ and *Atf4*^*−/−*^ mice were maintained on an HFD or control diet for 16 weeks, which has previously been shown to induce oxidative stress in the liver 30. We discovered that ATF4 expression was up-regulated in *ATF4*^*+/+*^ mice (Fig. 2A), suggesting that ATF4 is involved in HFD-induced oxidative stress. Moreover, HFD-induced oxidative stress significantly increases in the quantity of hepatic MDA and release of H_2_O_2_ in *Atf4*^*+/+*^ mice; however, only a mild increase was observed in the liver of *Atf4*^*−/−*^ mice (Fig. 2B and C), suggesting that ATF4 deficiency may ameliorate HFD-induced oxidative stress *in vivo*. As increased oxidative stress is a major contributor to the development of hepatic steatosis 31, we also examined fat content in the livers of both mice strains. Consistent with a previous study 6, HFD induced hepatic steatosis in *Atf4*^*+/+*^ mice, but not in *Atf4*^*−/−*^ mice, as demonstrated by oil red O and haematoxylin and eosin staining (Fig. 2D) and significant hepatic and serum TG accumulation in *Atf4*^*+/+*^ mice (Fig. 2E and F). Taken together, these results suggest that ATF4 deficiency protects mice from oxidative stress and TG accumulation in the liver induced by HFD.

To test the possibility that limiting lipid accumulation results in the inhibition of oxidative stress in *Atf4*^*−/−*^ mice, lipid metabolism genes were analysed by RT-PCR. Our results indicated that there was no difference in the expression of lipid metabolic genes, including lipogenic genes, lipid uptake genes, and genes related to lipid secretion (Fig. S2). These results also indicate that lipid metabolism gene regulation might not be a causal factor for protecting ATF4-deficient mice from HFD-induced triglyceride accumulation in the liver. Therefore, it is unlikely that inhibition of oxidative stress is the result of ATF4 deficiency limiting lipid accumulation in hepatocytes. Furthermore, ATF4 overexpression induced ROS production in wild-type mice (Fig. 4B), suggesting that ATF4 may directly regulate ROS production rather than lipid metabolism.

### CYP2E1 expression is much lower in the livers of *Atf4*^*−/−*^ mice compared with *Atf4*^*+/+*^ mice

**Figure 3:**
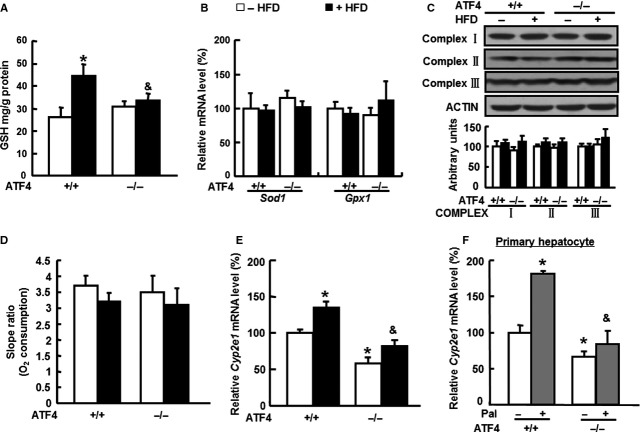
*Cyp2e1*mRNA levels are lower in the livers of *ATF4*^*−/−*^ mice compared with *ATF4*^*+/+*^ mice under high-fat diet (HFD). Six-week-old male *Atf4*^*+/+*^ (ATF4 + /+) and *Atf4*^*−/−*^ (ATF4 −/−) mice were fed HFD (+HFD) or normal diet (−HFD) for 16 weeks (*n* = 6). Reduced glutathione contents (A), *Sod1* and *Gpx1*mRNA levels (B), mitochondrial complex proteins levels (C), and oxygen consumption (D), and *Cyp2e1*mRNA levels (E) in the livers of mice were compared. *Cyp2e1*mRNA levels measured in primary hepatocytes following treatment with 0.2 mM Palmitate (+Pal) or vehicle (−Pal) (F). * indicates *P* < 0.05 as compared with the *Atf4*^*+/+*^/−HFD or *Atf4*^*+/+*^/−Pal group; & indicates *P* < 0.05 as compared with the *Atf4*^*+/+*^/+HFD or *Atf4*^*+/+*^/+Pal group. Data are the means ± SEM of two independent experiments.

Significantly higher levels of oxidative stress in the livers of *Atf4*^*+/+*^ mice fed a HFD are likely to reflect an imbalance between the production of ROS and the ability to detoxify the reactive intermediates by endogenous antioxidants such as GSH, SOD 32–33 and Glutathione peroxidase (GPX)1 34. We found that the levels of GSH were significantly increased by HFD in *Atf4*^*+/+*^ mice, but not in *Atf4*^*−/−*^ mice (Fig. 3A), and no differences were observed between the two strains in SOD levels (data not shown). Moreover, no differences were observed in the levels of *Sod1* and *Gpx1* mRNA in the livers of *Atf4*^*−/−*^ mice compared with *Atf4*^*+/+*^ mice (Fig. 3B). These results suggest that the protection against oxidative stress in *Atf4*^*−/−*^ mice is not because of the effects of increased anti-oxidative stress.

As mitochondrial dysfunction and overexpression of CYP2E1 are critical factors for determining free radical production in the mammalian liver 35–36, we examined whether the mitochondrial function in the livers was different between the two stains. We found that there was no difference in the levels of mitochondrial COMPLEX proteins (I–III) in the livers of *Atf4*^*+/+*^ and *Atf4*^*−/−*^ mice maintained on a HFD or ND (Fig. 3C). Additionally, the capacity of hepatocytes for oxygen consumption did not differ between the two mouse strains either on an ND or HFD (Fig. 3D). These results suggest that the protective effect of ATF4 deficiency against oxidative stress is independent of mitochondrial function.

We then examined *Cyp2e1* mRNA levels in the livers of *Atf4*^*+/+*^ and *Atf4*^*−/−*^ mice maintained on an ND or HFD diet. We discovered that the basal expression of *Cyp2e1* mRNA levels was lower in *Atf4*^*−/−*^ mice when compared with their *Atf4*^*+/+*^ counterparts, and a significant induction was observed in *Atf4*^*+/+*^ mice when on a HFD, this was not observed in *Atf4*^*−/−*^ mice (Fig. 3E). The effect of low CYP2E1 expression on oxidative stress induced by ATF4 deficiency was further investigated in primary hepatocytes isolated from *Atf4*^*+/+*^ and *Atf4*^*−/−*^ mice following palmitate treatment, which has been shown to induce ROS production in *Atf4*^*+/+*^, but not in *Atf4*^*−/−*^ mice (Fig. 1B). Consistent with our *in vivo* observations, we found that palmitate treatment significantly increased the levels of *Cyp2e1* mRNA in primary hepatocytes isolated from *Atf4*^*+/+*^, but not *Atf4*^*−/−*^ mice, and the basal levels of *Cyp2e1* mRNA were also lower in the primary hepatocytes from *Atf4*^*−/−*^ mice (Fig. 3F). These results suggest the possible involvement of CYP2E1 in the protection against oxidative stress caused by ATF4 deficiency.

### ATF4 regulates CYP2E1 expression by enhancing CREB phosphorylation, which directly stimulates the CYP2E1 promoter activity

**Figure 4:**
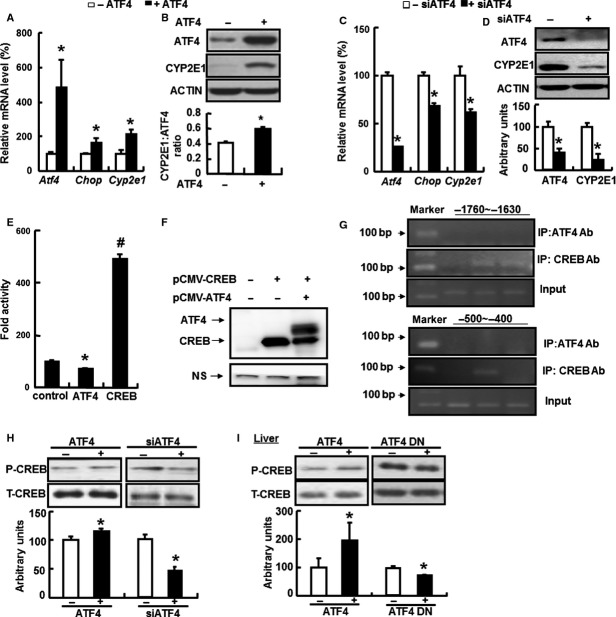
Effects of ATF4 on *Chop* and *Cyp2e1*mRNAs, CYP2E1 promoter activity and CREB phosphorylation. (A and B) HepG2 cells adenoviral transduction to overexpress ATF4 (+ATF4) or treatment with an empty adenovirus (−ATF4). (C and D) Primary hepatocytes were treated with siRNA ATF4 (+siATF4) or transfection reagent only (−siATF4). (E) Plasmids overexpressing ATF4 (pCMV-ATF4), CREB (pCMV-CREB) or pCMV-HA (control), and the CYP2E1 promoter (CYP2E1-P2600) were co-transfected in 293T cells and the promoter activity of CYP2E1 was analysed. (F and G) 293T cells were transfected with vectors overexpressing ATF4 (pCMV-ATF4), or CREB (pCMV-CREB), or both. Data are the mean ± SEM of at least four independent experiments. * effects of (+ATF4) group *versus* (−ATF4) group (two-tailed Student’s *t*-test, *P* < 0.05), # effects on (+CREB) group *versus* (+ATF4) group (two-tailed Student’s *t*-test, *P* < 0.05). (H) Primary hepatocytes were infected with adenovirus overexpression of ATF4 (+ATF4) or blank adenovirus (−ATF4), and siRNA ATF4 (+siATF4) or transfection reagent only (−siATF4). (I) Mice were injected with adenovirus overexpression of ATF4 (+ATF4) or an empty adenovirus (−ATF4), and an ATF4 dominant negative (+ATF4 DN) or control green fluorescent protein adenovirus (−ATF4 DN). (A and C) *Atf4*,*Chop* and *Cyp2e1*mRNAs; (B and D) ATF4 and CYP2E1 protein levels (*top*, western blot; *bottom*, quantitative measurements of ATF4 and CYP2E1 protein relative to ACTIN); (E) CYP2E1 promoter activity; (F) overexpression of CREB (pCMV-CREB), or ATF4 (pCMV-ATF4), or both; (G) Comparison of ATF4 and CREB binding at CRE site with overexpression of CREB (pCMV-CREB), or ATF4 (pCMV-ATF4), or both by ChIP assay; (H and I) phosphorylation of CREB (P-CREB) in primary hepatocytes in (H) and livers in (I) (*top*, western blot; *bottom*, quantitative measurements of P-CREB protein relative to their total protein). * indicates *P* < 0.05 as compared with the control [(−ATF4), or (−siATF4), or (−ATF DN), or (control)] group. # indicates *P* < 0.05 as compared with control group. Data are the means ± SEM of two independent experiments.

We next examined whether ATF4 regulates CYP2E1 levels in HepG2 cells infected with adenovirus expressing ATF4 (Ad-ATF4) or blank adenovirus (Ad-Null). The effects of Ad-ATF4 were validated by the significant increase in levels of *Atf4* and *Chop* mRNAs (Fig. 4A). *Cyp2e1* mRNA and CYP2E1 protein were significantly increased with the overexpression of ATF4 compared with the Ad-Null group (Fig. 4A and B). Conversely, mRNA levels of *Atf4*,*Chop* and *Cyp2e1*, and CYP2E1 protein were significantly reduced in primary hepatocytes when ATF4 expression was attenuated *via* siRNA treatment (Fig. 4C and D).

It has been shown that there is a cAMP-responsive element (CRE) binding site at the CYP2E1 promoter, suggesting that ATF4 may regulate CYP2E1 expression by directly binding to this site. In contrast to a stimulatory effect of ATF4 on CYP2E1 expression, the CYP2E1 promoter was unexpectedly repressed by ATF4 overexpression (Fig. 4E), suggesting that ATF4 indirectly regulates CYP2E1 expression. Activating transcription factor 4 belongs to the same family as CREB and therefore has been shown to regulate expression of its target genes *via* binding at CRE site, and thus has many biological functions 37. Moreover, this raises the possibility that CREB might be involved in this regulation. Therefore, we examined the effect of CREB on CYP2E1 promoter activity and found that overexpression of CREB significantly stimulates CYP2E1 promoter activity (Fig. 4E). To determine the possibility of CREB or ATF4 binding to the potential CRE sites including −1702 to −1695 (-tgtcctca-) and −453 to −445(agacatca), only CREB or both CREB and ATF4 were overexpressed in 293T cells for CHIP analysis. We were able to successfully overexpress both CREB and ATF4 (Fig. 4F). CHIP analysis revealed that −1760 to 1630 and −500 to −400 sequences, which are located in the CYP2E1 promoter, were significantly increased when CREB was overexpressed. The −500 to −400 sequence specifically decreased with ATF4 and CREB co-expression in cells (Fig. 4G). Moreover, ATF4 did not bind to potential CRE sites in the CYP2E1 promoter when ChIP was performed by using anti-ATF4 antibodies (Fig. 4G). These results suggest that CREB can directly regulate CYP2E1 expression, and that ATF4 inhibits CYP2E1 expression by preventing the binding of CREB to the CRE site (−500 to −400) located in the CYP2E1 promoter. The added chromatin revealed a similar numbers of bands when amplifying both regions indicated as Input (Fig. 4G).

To investigate the possibility that CREB may mediate ATF4 regulation of CYP2E1 expression, we examined the effect of ATF4 overexpression and knock-down on CREB phosphorylation levels in primary hepatocytes. As expected, we found that CREB phosphorylation was significantly increased or decreased by ATF4 overexpression or knock-down, respectively (Fig. 4H). Consistent with these results, a similar regulatory pattern was observed in the livers of mice injected with the adenovirus that results in the overexpression or knock-down ATF4 expression (Fig. 4I). Taken together, these results suggest that ATF4 regulates CYP2E1 expression in a CREB-dependent manner and that CREB directly binds to the CYP2E1 promoter.

### ATF4 increases ROS production *via* increasing CYP2E1 expression

**Figure 5:**
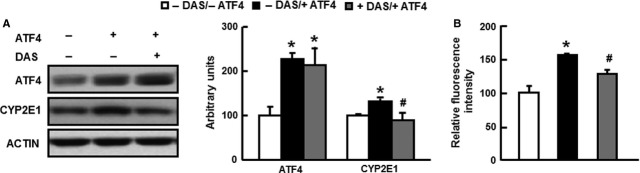
Overexpression of ATF4 increases CYP2E1 expression and ROS production *in vivo*. Eight-week-old male *Atf4*^*+/+*^ mice were injected with adenovirus overexpressing ATF4 (+ATF4) or blank adenovirus (−ATF4) as control in the presence (+DAS) or absence (−DAS) of diallyl sulphide (DAS, 200 mg/kg, i.p, daily), a selective CYP2E1 inhibitor (*n* = 4). (A) ATF4 and CYP2E1 protein levels in livers; (B) ROS production measured in primary hepatocytes. * indicates *P* < 0.05 as compared with the - DAS/−ATF4 group; # indicates *P* < 0.05 as compared with the – DAS/+ATF4 group. Data are the means ± SEM of two independent experiments.

Given the importance of ATF4 in regulating CYP2E1 expression in hepatocytes *in vitro*, we hypothesized that the protection against HFD-induced oxidative stress in the livers by ATF4 deficiency is possibly mediated by inhibiting CYP2E1 expression. To test this hypothesis, we examined the inhibitory effect of DAS, a selective inhibitor of CYP2E1 38, on ROS production in mice injected with an adenovirus to overexpress ATF4. As expected, overexpression of ATF4 stimulated CYP2E1 expression and ROS production, which were significantly blocked by the injection of DAS (200 mg/kg, i.p) once a day for 7 days (Fig. 5A and B). These results suggest that the stimulating effects of ATF4 on oxidative stress in liver are mediated *via* increased expression of CYP2E1.

### Reduced ATF4 expression in the liver protects mice from HFD-induced oxidative stress, *CYP2E1 expression*, and TG accumulation

**Figure 6:**
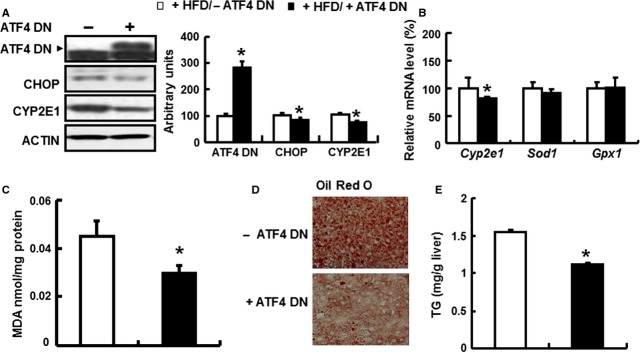
Knocking down ATF4 expression in liver protects mice from high-fat diet (HFD)-induced oxidative stress and TG accumulation. (A–E) Six-week-old mice were fed HFD for 1 week, then injected with adenovirus expressing dominant negative ATF4 (+ATF4 DN) or Ad-GFP (−ATF4 DN) and maintained on a HFD (+HFD) for two more weeks (*n* = 4). (A) ATF4 DN, CHOP, and CYP2E1 proteins (*right*, western blot; *left*, quantitative measurements of ATF4 DN or CHOP or CYP2E1 protein relative to ACTIN); (B) *Cyp2e1*,*Sod1* and *Gpx1*mRNA levels, (C) MDA levels, (D) oil red O staining and (E) TG content in livers were compared. * indicates *P* < 0.05 as compared with the control (+HFD/−ATF4 DN) group. Data are the means ± SEM of two independent experiments.

To examine whether ATF4 expression in the liver is responsible for protecting against HFD-induced oxidative stress, CYP2E1 expression, and TG accumulation, adenovirus expressing dominant-negative ATF4 (Ad-ATF4 DN) or GFP (Ad-GFP) were injected into mice maintained on an HFD for 1 week, followed by a continuous HFD for further 2 weeks, a period that has been shown is sufficient to increase *Cyp2e1* expression in livers 39. Following injection with Ad-ATF4 DN (as confirmed by western blotting), expression levels of its downstream target CHOP 40 were decreased significantly in the livers of these mice compared with the Ad-GFP group (Fig. 6A). As expected, we found that knocking down expression of ATF4 in liver prevents HFD-induced *Cyp2e1* expression (Fig. 6A). No difference in the levels of *Sod1* and *Gpx1* mRNA, however, was observed between the two groups (Fig. 6B). MDA levels were also significantly reduced by ATF4 deletion (Fig. 6C). Consistent with these observations, ATF4 deletion also significantly attenuated TG accumulation as demonstrated by oil red staining (Fig. 6D) and TG measurement (Fig. 6E).

## Discussion

ATF4-deficient mice are resistant to HFD-induced NAFLD. In this work, the results showed that ATF4 deficiency has no effect on expression of lipid metabolic genes including lipogenic genes, lipid uptake genes, and lipid secretion-related genes (Fig. S2). In addition, GRP78, the marker of ER stress, exhibits no difference in liver of *Atf4*^*−/−*^ mice compared with wild-type mice either fed on a control diet or an HFD diet, suggesting that ER stress is not causal factors for ATF4-deficient protecting against HFD-induced NAFLD (Fig. S3A). Although ATF4 directly regulates FGF21 expression, the result showed no difference in expression of FGF21 in the liver of *Atf4*^*−/−*^ mice compared with wild-type mice both fed on an HFD diet (Fig. S3B). All these results suggested that ATF4 deficiency protecting mice from HFD-induced NAFLD does not depend on the function of ATF4 in regulation of lipid metabolism, ER stress, or FGF21 expression.

Activating transcription factor 4 has been shown to regulate oxidative stress in a cell type-specific manner 7,8. In contrast to a previous study showing that ATF4-deficient fibroblasts are prone to oxidative stress 7, we found that deletion of ATF4 was protective against oxidative stress in hepatocytes *in vitro* and *in vivo*. Our results are consistent with previous work demonstrating similar effects of ATF4 on regulating oxidative stress in neurons 8 and HEK293 cells 9. The different roles ATF4 plays in regulating oxidative stress in different cell lines or tissues suggest the complexity in understanding its role in regulating oxidative stress under different conditions. Furthermore, it has been shown that ATF4 functions as a transcriptional repressor in many cases 41–42. Importantly, this work indicates that ATF4 may also function as a transcriptional activator in hepatocytes.

Chronic HFD feeding is characterized by the presence of oxidative stress 43–44. In this study, we found that HFD feeding for 16 weeks results in significantly increased expression of ATF4 in liver of *Atf4*^*+/+*^ mice. Moreover, as marker of oxidative stress, the levels of MDA and H_2_O_2_ release were also increased in response to HFD, and this is blocked with deficient ATF4 levels. These results suggest that ATF4 is involved in the regulation of HFD-induced oxidative stress. Furthermore, we provided evidence that ATF4 expression in the liver is responsible for these changes, as similar blocking effects on HFD-induced oxidative stress and CYP2E1 expression were obtained in mice by using adenovirus-mediated ATF4 knock-down in liver.

Although the function of pro-oxidative stress of ATF4 in hepatocytes is consistent with that in neurons 8, the underlying mechanisms may be different. Redox homoeostasis is maintained by a balance between the rates of production and clearance of free radicals 45. A disturbance of this balance such as overproduction of ROS and/or decrease in anti-oxidative capacity normally results in oxidative stress and cellular injury. In neurons, the resistance to oxidative stress by ATF4 deletion was associated with a decreased clearance of the antioxidant GSH 8. The protective effects of ATF4 deficiency against oxidative stress in hepatocytes, however, are not because of the increase in antioxidative capability as evidenced by the contents of antioxidative enzymes such as GSH and SOD. These results suggest the possibility of tissue-specific mechanisms underlying the protection against oxidative stress caused by ATF4 deficiency.

Mitochondrial dysfunction and increased CYP2E1 activity are major contributors to ROS overproduction 35–36. However, our data show that an ATF4 deficiency does not alter mitochondrial function. Therefore, the protection against oxidative stress by ATF4 knock-down is not dependent on mitochondrial function in hepatocytes. The low levels of CYP2E1 in *Atf4*^*−/−*^ mice on either a ND or HFD highlight the potential association between ATF4 and CYP2E1, which has been demonstrated by ATF4 regulation of CYP2E1 *in vitro*. Moreover, the mitigation of ROS production by the potent CYP2E1 inhibitor in mice further emphasized the involvement of CYP2E1 in ATF4-dependent ROS production in the context of overexpression of ATF4 by adenoviruses. Nevertheless, a more recent study shows that both CYP2E1 and alcohol can induce the expression of ATF4 and the integrated stress response in hepatocytes 46. The discrepancy in the relationship of ATF4 and CYP2E1 between their study and ours highlights the complexity of CYP2E1 in ATF4-related stress response. While being inconclusive, we can deduce that a cooperative positive feedback might exist between ATF4 and CYP2E1 and that this feedback can be enhanced by external factors such as alcohol and an HFD, *etc*.

In this study, we found that ATF4 is an important regulator for CYP2E1 expression in hepatocytes. Although a CRE binding site is identified in the CYP2E1 promoter, we did not observe a direct effect of ATF4 on CYP2E1 expression. Our results suggest that CREB is involved in the regulation of CYP2E1 by ATF4, as demonstrated by the fact that overexpression of CREB directly stimulates the CYP2E1 promoter *via* binding to the potential CRE site located in the −1760 to 1630 and −500 to −400 sequences. Furthermore, ATF4 can inhibit CREB binding to the sequence of −500 to −400 to regulate the CYP2E1 expression. Moreover, CREB phosphorylation was also regulated by ATF4 overexpression or knock-down. Taken together, these results suggest that ATF4 regulates CYP2E1 expression in a CREB-dependent manner that can directly stimulate the CYP2E1 promoter activity.

To demonstrate the importance of ATF4 expression in the liver and regulation of HFD-induced oxidative stress, we mitigated ATF4 expression by using an adenoviral system in the liver of mice *via* the Ad-ATF4 DN adenovirus while being maintained on a HFD for 3 weeks. During this time frame, the HFD feeding induces oxidative stress *via* increasing expression of CYP2E1 39–47. Consistent with a role for liver ATF4 in the regulation of HFD-induced oxidative stress, we found that MDA levels and TG accumulation were largely blocked by the Ad-ATF4 DN treatment of mice *via* inhibiting the *Cyp2e1* expression.

In conclusion, our current results indicate that ATF4 is a pro-oxidative stress transcription factor that is expressed in the liver in response to HFD, and probably inhibits ROS production by modulating of CYP2E1 expression. Moreover, we showed that ATF4-regulated CYP2E1 expression occurs in a CREB-dependent manner, and that this could directly stimulate the CYP2E1 promoter activity. Our study also provides evidence for ATF4 being a potential novel therapeutic target to treat disease related to oxidative stress.

## Supporting information

Figure S1 ATF4 deficiency protects primary hepatocytes from palmitate-induced ROS production.

Figure S2 ATF4 deficiency has no effects on the expression of lipid metabolic genes. (A-C).

Figure S3 ATF4 deficiency has no effects on the expression of GRP78 and FGF21.
